# High Burden of *Chlamydia trachomatis* and Human Papillomavirus Infections in Low-Income Female University Students from Public Schools in the Brazilian Amazon

**DOI:** 10.3390/microorganisms14061176

**Published:** 2026-05-23

**Authors:** Leonardo Miranda dos Santos, Rodrigo Covre Vieira, Louise de Souza Canto Covre, Milena Cristina Martins da Silva, Thiago de Matos Bezerra, Geraldo Mariano Moraes de Macedo, Edna Aoba Yassui Ishikawa, Karla Valéria Batista Lima, Maísa Silva de Sousa, Rodrigo Vellasco Duarte Silvestre

**Affiliations:** 1 Laboratory of Molecular and Cellular Biology, Center for Tropical Medicine, Federal University of Para, Belem 66075-110, PA, Brazil; rodrigobjt@hotmail.com (R.C.V.); louisecanto12@yahoo.com.br (L.d.S.C.C.); milenacristinamartins8080@gmail.com (M.C.M.d.S.); thiagodematos50@gmail.com (T.d.M.B.); ishikawaufpa@gmail.com (E.A.Y.I.); maisasousa@ufpa.br (M.S.d.S.); 2 State Department of Education of Pará, Government of the State of Pará, Belem 66020-080, PA, Brazil; 3 Laboratory of Tropical Dermatology, Center for Tropical Medicine, Federal University of Para, Belem 66075-110, PA, Brazil; geraldo_mmm@yahoo.com.br; 4 Bacteriology and Mycology Section, Evandro Chagas Institute, Ananindeua 67030-000, PA, Brazil; karlalima@iec.pa.gov.br; 5 Papillomavirus and Retrovirus Laboratory, Virology Section, Evandro Chagas Institute, Ananindeua 67030-000, PA, Brazil; rodrigosilvestre@iec.gov.br

**Keywords:** *Chlamydia trachomatis*, HPV, students, Amazon, public health

## Abstract

Sexually transmitted infections (STIs) caused by *C. trachomatis* and HPV are the most prevalent worldwide. College students are characterized by being young women of reproductive age who may have risky sexual behavior. To describe the prevalence and factors associated with endocervical infection by *C. trachomatis* and HPV in college women in the Brazilian Amazon. Endocervical secretions were collected. The *ompA* gene of *C. trachomatis* and the *L1* gene of HPV were detected. The Chi-square test, Fisher’s exact test, G test, Odds Ratio, and Multiple Logistic Regression were used with 95% confidence interval and *p* ≤ 0.05. The overall prevalence of endocervical infection by *C. trachomatis* was 8.3% (25/302) and by HPV was 28.9% (87/302). Low income was associated with sexually transmitted infection by *C. trachomatis* (14.8%, *p* = 0.0336). Those under 25 years old had twice the chance of HPV infection [39.3%, (OR: 2.6989), 95% CI: 1.6054–4.5371, *p* = 0.0002], as did women without children [31.8%, (OR: 2.333), CI: 1.1235–4.8461, *p* = 0.0307]. Women who did not study in a public school had 63% reduced risk of acquiring HPV infection [45.8% (OR: 0.3713), CI: 0.1951–0.7064, *p* = 0.0035]. *C. trachomatis* and HPV infections were present in low-income, childless young women who attended public schools, requiring the intensification of STI prevention policies in the Amazon region.

## 1. Introduction

Sexually transmitted infections (STIs) caused by *Chlamydia trachomatis* and human papillomavirus are serious global health issues, as they are prevalent infections with widespread exposures levels in the world’s population, becoming a challenge for screening schemes, with 128.5 million annual cases of *C. trachomatis* and a global prevalence of 31% for HPV [1,2]. Endocervical infection by *C. trachomatis* increases the persistence of HPV infection and its genetic materials may be present in 99% of cervical cancers [3,4], in addition to facilitating HIV infection [5].

*C. trachomatis* is an obligate intracellular bacterium classified into 19 genotypes according to the variability profile in its *ompA* gene, with genotypes A to C causing trachoma, genotypes L1 to L3 causing lymphogranuloma venereum, and genotypes D to K causing endocervical infection that is asymptomatic in up to 80% of women [6]. Infections by *C. trachomatis* can progress in up to 19.7% of cases to Pelvic Inflammatory Disease (PID) and subsequent permanent damage, such as infertility and ectopic pregnancies [7,8,9]. HPV is a non-enveloped virus of the Papillomaviridae family that infects the skin and mucous membranes of vertebrates. Based on the similarity of its *L1* gene, there are more than 200 types divided into five groups (α, ß, γ, µ and ν) and the high oncogenic risk are HPV16, 18, 31, 33, 35, 39, 45, 51, 52, 56, 58, and 59, with HPV 16 and 18 being present in 70% of invasive cervical cancer cases [10]. HPV infection is responsible for a spectrum of atypia and cytopathological lesions that favor cervical squamous cell carcinoma as Low-Grade Squamous Intraepithelial Lesions (LSILs), including Atypical Squamous Cells of Undetermined Significance (ASC-US) and CIN grade 1 (CIN1), High-Grade Intraepithelial Lesions (HSILs), and cervical carcinoma in situ [11,12].

The Brazilian health system offers the Pap smear test and HPV vaccination as the main measures for preventing cervical cancer. The DNA-HPV screening program and genotyping of oncogenic HPV types, although promising, is being discreetly introduced into public primary health care [13,14]; however, any health policies for *C. trachomatis* screening in the asymptomatic young population have not been established [15]. In Brazil, the prevalence of *C. trachomatis* varies from 2.2% to 10.8% [16,17,18,19,20,21] and that of HPV is 25.4% [22]. In the Amazon, the frequency of HPV varies from 15.5% to 63.3%, the highest rates of cervical cancer in the national territory [23,24,25,26]. University women in the Amazon region are characterized by being young women of reproductive age most likely to have risky sexual behavior and socioeconomic vulnerability and/or come from remote communities with poor access to higher education and gynecological health services [27,28,29]. In countries with very specific public screening and molecular diagnosis programs, preventive issues are well targeted when the profile of each population is considered. Knowledge of the prevalence and factors associated with endocervical infection by *C. trachomatis* and HPV in college women is important to understand the epidemiology of this infection and to plan prevention strategies targeted at this population. This study aimed to describe the prevalence and factors associated with endocervical infection by *C. trachomatis* and HPV in female students at the largest public university in the Brazilian Amazon region.

## 2. Materials and Methods

### 2.1. Type and Variables of Study

This is a pilot, cross-sectional analytical study that lasted from May 2018 to January 2020. Asymptomatic female university students, aged 18 or over, spontaneously attended by the Cytopathology Laboratory and Clinical Analysis Laboratory (LAC) of the Institute of Biological Sciences (ICB) of the Federal University of Para (UFPA), were invited to participate in the study. The inclusion criteria were female students regularly enrolled in undergraduate and graduate courses at the Federal University of Para, over 18 years old, who had initiated sexual activity. The exclusion criteria were menstruating or pregnant women, students who had undergone partial or total hysterectomy or with history of serious cervical injuries, in addition to those who did not wish to participate in the project and/or did not sign the free and informed consent form (FICF). The variables of this study were as follows: municipality of origin, age (years), marital status, family income (Brazilian minimum wages), number of children, alcohol use, having a public school background in basic education, coitarche (years), number of sexual partners in life, constant condom use, miscarriage, menarche, cytological conditions (LSISL/ASCUS, HSIL/ASC H, Normal), Pap test frequency, and HPV vaccination.

### 2.2. Collection and Analysis of Biological Sample (Endocervical Secretion)

Endocervical secretions were collected during the Pap smear using an endocervical brush, Ayres spatula, and a glass slide for subsequent analysis. The samples were stored in cryogenic tubes with 1 mL of Tris-EDTA (TE) buffered solution [10 mM Tris-HCl pH 8.5; 1 mM EDTA] at a temperature of −20 °C. During the Pap smear, the ectocervix and endocervix were scraped, the collected material was transferred to a glass slide previously identified and fixed with alcohol (96%). The usual Papanicolaou staining (Harris hematoxylin, Orange G and EA-36) was performed and subsequently analyzed by light microscopy. The results were classified as normal or atypical cells (atypia). Atypical cells of undetermined significance are divided according to their origin site: glandular (AGG) or squamous (ASC). Cells of squamous origin were also subdivided into Low-Grade Intraepithelial Lesion (LSIL, comprising HPV cytopathic effect and CIN I), high-grade intraepithelial lesion (HSIL, comprising CIN II and CIN III) and atypical squamous cells (ASCs). The high-grade lesions where microinvasion and carcinoma could not be excluded were classified as invasive squamous cell carcinoma. Glandular lesions were divided into adenocarcinoma in situ (AIS) and invasive adenocarcinoma. Conventional cytology results were classified using Bethesda terminology [30].

#### 2.2.1. DNA Extraction

Genomic DNA extraction was performed using a pureLink genomic DNA purification kit (Invitrogen, Carlsbad, California, USA), following the manufacturer’s recommendations, and then, the samples were stored at −20 °C until further analysis. A Polymerase Chain Reaction (PCR) of the *human β-globin* gene was performed before detection of *C. trachomatis* and HPV to confirm sample suitability.

#### 2.2.2. Amplification of the *human β-globin* Gene

All genomic DNA extracted was subjected to a PCR for amplification of a 268 bp fragment of the *human β-globin* gene to verify the quality of the extracted DNA. For this step, PC03/GH20 primers were used. The reaction consisted of 5 µL of GoTaq Green Master Mix (Promega Biotecnologia, Madison, Wisconsin, USA), 2.5 µL of H_2_O, 0.25 µL of each primer (0.5 µM), and 2 µL of extracted DNA. The temperature cycles had an initial denaturation at 94 °C for 4 min, followed by 35 cycles at 94 °C for 45 s, annealing at 55 °C for 45 s, and extension at 72 °C for 45 s, with a final extension at 72 °C for 8 min. PCR products were electrophoresed on a 2% agarose gel in 1× TAE buffer (100 V, 1 h), stained with Sybr Safe (4 µL), and visualized under ultraviolet light. Sterile water was included as a negative control to rule out contamination.

#### 2.2.3. Detection of an *ompA* Gene Fragment from *C. trachomatis*

For the detection of endocervical infection by *C. trachomatis*, we performed a modified nested-PCR protocol [31], which amplifies a 394 pb pair fragment of the chromosomal *ompA* gene of *C. trachomatis*. For the first nested-PCR, we used 6.0 μL of GoTaq Green Master mix (Promega, Madison, WI, USA), 0.5 μL of primers P1 (A) (5′GACTTTGTTTTCGACCGTGTT-3′), and P2 (5′AGCRTATTGGAAAGAAGCBCCTAA-3′) with a concentration of 20 pmol/μL of each primer; in addition, we used 2 μL of genomic DNA and 3 μL of sterile water, resulting in a final volume of 12 μL. In the second nested-PCR, we used 0.5 μL of the amplified material from the first nested-PCR, 6.0 μL of Go Taq Green Master Mix (Promega, Madison, WI, USA), 4.5 μL of sterile water and 0.5 μL (20 pmol/μL) of primers P3 (5′-AAACWGATGTGAATAAAGARTT-3′) and P4 (5′-TCCCASARAGCTGCDCGAGC-3′). Positive and negative controls were used in all reactions to optimize the results of choosing the positive sample and to verify DNA contamination between the samples, respectively. In all nested-PCR, an initial temperature of 95 °C was considered, lasting five minutes in the first nested-PCR and 1 min in the second nested-PCR, followed by 35 cycles of denaturation at 94 °C for 40 s, annealing at 54 °C for 30 s and extension at 72 °C for 90 s, with a final extension step at 72 °C for 7 min. The amplified products were visualized by electrophoresis in a 1% agarose gel with 0.5 mg/mL ethidium bromide.

#### 2.2.4. Detection of HPV *L1* Gene Fragment

To detect HPV infection, we used the degenerate primers MY09/MY11, which amplify a fragment of the HPV *L1* gene and have between 449 pb and 458 pb, depending on the HPV type. The sequences of the primers used were as follows: MY09 (5′-CGTCCMAARGGAWACTGATC-3′) and MY11 (5′-GCMCAGGGWCATAAYAATGG-3′), with the following correspondents: M = A + C, R = A + G, W = A + T, Y = C + T. For this step, 0.2 mL microtubes were used in an Applied Biosystems 2720 thermocycler. The final reaction volume was 10 µL [5 µL of Go Taq Green Master Mix 2X (CF = 1x), 1 µL of each primer (10 µM; CF = 1 µM), 2 µL of nuclease-free H_2_O and 2 µL of DNA]. The PCR followed the steps of initial denaturation at 94 °C for 2 min, followed by 35 cycles with denaturation at 94 °C for 45 s, primer hybridization at 55 °C for 45 s and chain extension at 72 °C for 45 s. Finally, the material was kept at 72 °C for 10 min (final extension). The positive control was a known positive sample (HPV16) and the negative control was nuclease-free water [32].

### 2.3. Ethical Aspects

The investigations were conducted in accordance with point 23 of the Declaration of Helsinki (1975, revised in 2013) and the Resolution 466/2012 of the National Health Council [33]. This study is part of the project “Detection and genotyping of *C. trachomatis* in university students treated at the cytopathology laboratory/UFPA: cytological and molecular analysis”, considering all regulations to ensure the ethics and confidentiality of the participants. Our research was authorized by the Research Ethics Committee of the Center for Tropical Medicine of the Federal University of Para (Registration: 103,571/CAAE 07821212,2,0000,5172) Approved on 19 September 2012. Only college women over 18 years old who read and signed the free and informed consent form (ICF) before the collection of biological samples and socioepidemiological and gynecological data were included in this study. All data were analyzed with full anonymity. Participants who tested positive for sexually transmitted infection with *C. trachomatis* were referred for medical evaluation.

### 2.4. Statistical Analysis

The Statistical Package for Social Sciences (SPSS) version 21.0 (SPSS, Chicago, IL, USA) was used for the analyses. The chi-square, Fisher’s exact, and Odds Ratio tests were used to analyze categorical variables with only two options. The G test of independence was used for variables with more than two options in all our analyses of comparisons between the variables and the positivity of *C. trachomatis* and HPV infection. For variables with more than two options, the G test of independence was used along with Cramer’s V test to verify the effect size of these variables, in which the level of association was defined as weak (less than or equal to 0.29), moderate (0.30 to 0.49), or strong (0.50 or greater). Multiple logistic regression was used to reduce the chances of bias. We considered for this study a 95% confidence interval (CI) and a significance level of *p* ≤ 0.05.

## 3. Results

**Figure 1:**
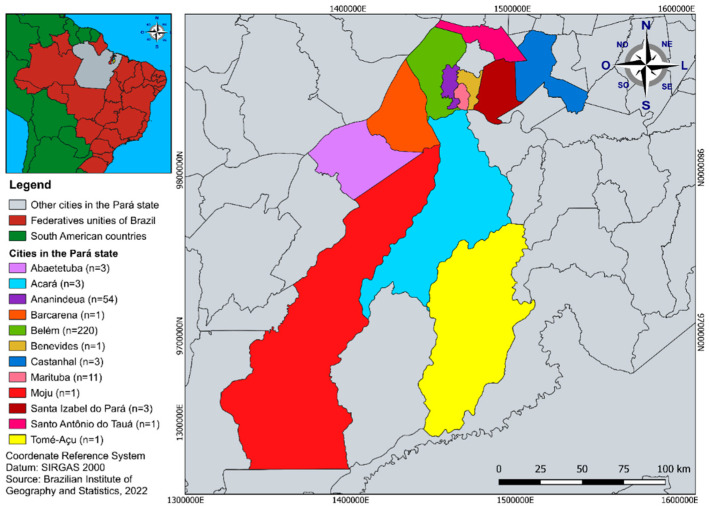
Number of college women by their respective municipalities of origin in the state of Para, Amazon region of Brazil.

In this study, 52% (157/302) of the participants were 25 years old or older, 90.7% (274/302) were single, 72.2% (218/302) had a family income of one to three Brazilian minimum wages, 80.1% (242/302) had no children, 60.6% (183/302) consumed alcohol, 84.1% (254/302) had a public school education, 76.2% (232/302) had sexual life after 15 years of age, 84.1% (254/302) had more than one sexual partner in their lifetime, 72.9% (220/302) did not use condoms during sexual intercourse, 88.7% (268/302) had never had miscarriage, and 96.7% (292/302) had menarche at an age greater than 15 years (Table 1). In this study, the number of participants per municipality of origin was variable, concentrating in the capital Belém (N = 220), and in the interior: Ananindeua (*n* = 54), Marituba (*n* = 11), Abaetetuda (*n* = 3), Castanhal (*n* = 3), Acará (*n* = 3), Santa Izabel (*n* = 3); there was also one participant from each of these municipalities: Barcarena, Benevides, Moju, Santo Antônio do Tauá and Tome-Açu (Figure 1).

The overall prevalence of endocervical infection by *C. trachomatis* was 8.3% (25/302) and by HPV was 28.9% (87/302) in our entire study population. Cases of coinfection were 3.3% (10/302), all from women living in the capital (Table 1).

The participants’ median age was 25 years old (interquartile range: 22.0–29.25 years, range: 18–55 years). Among the female undergraduates who tested positive for endocervical infection by *C. trachomatis* and HPV, the median age was 23 years old (interquartile range: 21–26 years, range: 18–51 years) and 23 years old (interquartile range: 21–26 years, range: 18–55 years), respectively. Women who reported earning less than one Brazilian minimum wage were significantly associated with sexually transmitted infection by *C. trachomatis* (14.8%, *p* = 0.0497). College women under 25 years old were twice as likely to acquire HPV infection [39.3%, (OR: 2.6989), CI95%: 1.6054–4.5371, *p* = 0.0002], as were women who reported not having children [31.8%, (OR: 2.333), CI: 1.1235–4.8461, *p* = 0.0307]. Being a college student who did not attend a public school during basic education reduced the chances of acquiring this STI by 63% compared to those who came from a public school [45.8% (OR: 0.3713), CI: 0.1951–0.7064, *p* = 0.0035]. There was no significant difference between the prevalence of endocervical infections by *C. trachomatis* and HPV in students from the capital and those from the interior of Para (Table 2).

We did not observe a significant association between the variable’s cytological conditions, Pap smear performance and HPV vaccination; however, we observed that HSIL/ASC-H was the most common cytological condition among HPV-positive participants (54.5%) (Table 3).

## 4. Discussion

In this study, a high prevalence of endocervical infection by *C. trachomatis* (8.3%) was found in female students at the largest public university in the Amazon region of Brazil. Brazil’s universal health system has not yet created a screening policy for *C. trachomatis* in the asymptomatic young female population under 25 years old [15], so we do not know how to measure the total number of cases at the national level. However, the prevalence of *C. trachomatis* in Brazil ranges from 2.2% to 10.8% [16,17,18,19,20,21] and similar prevalence rates have been found in university students in the Brazilian Amazon [29] and in other cities such as Mbeya-Tanzania (11%) [34], Wisconsin, United States (7.2%) [35], and in Uppsala, Sweden [36] which, like in our study, report that socioeconomic vulnerability generated by poverty supports a large part of the social risk conditions for acquiring STIs caused by *C. trachomatis* [37].

In Brazil, gynecological health services in primary care operate with great logistical, structural and financing difficulties for the low-income population [28], and the impacts of this situation can be observed in the high rates of hospitalization for PID [38,39]; this is probably the reason why the participants who had a family income below the Brazilian minimum wage (equivalent to USD 250) were significantly associated with *C. trachomatis* infection (*p* = 0.0497). Public universities in the Amazon are an open space that receives a great diversity of people from different social standards; about 70.2% of them are composed of low-income people [40] and many of them receive financial aid to support themselves in the capital [41]. This study showed high prevalence of endocervical HPV infection (28.9%) comparable with the national HPV burden, which is 25.4% to 54% [22,42], as well as the burden in college women in Brazil [22,27,43] and in university students from Maputo, Mozambique (28.6%) [44], Vietnam (4.0%) [45], and Gaborone, Botswana (31%) [46]. HPV rates from 15.5% to 63.3% have been detected in the Amazon [9,17,21] and even during the establishment of public measures to control and prevent this infection, this region has the highest number of cases of cervical cancer and low anti-HPV vaccine adherence [47].

Young age is a major risk factor for STIs, as it is a phase of life in which women acquire new perceptions, with changes in feelings and the search for new experiences to form their personal identity. These aspects interfere in their sexual life, and high rates of STIs are often associated with risky sexual behavior [48]. In the present study, the results showed that university girls under 25 years old were twice as likely to acquire the HPV infection (OR: 2.6989, *p* = 0.0002). Childless women were twice as likely to acquire endocervical HPV infection compared to those with children (OR: 2.333, *p* = 0.0307), which is a consequence of the risky sexual behavior admitted by these young women, who, because they do not have children, and possibly not even a stable relationship and/or steady partner, feel free to experiment variable sexual practices and new partners [49]. In this case, sexually active young college women may be unassisted by public preventive health services and health education on STIs, unlike women with children who generally have a steady sexual partner and had preventive medical monitoring for HPV during pregnancy in prenatal care [50,51].

Sexual education about STIs and vaccination against HPV are mandatory for adolescents and young adults in primary and secondary schools in Brazil, through the Brazilian National Curricular Guidelines for Education [52] and parallel actions by the Brazilian Government through the Health in School Program [53].

In our study, college women who did not come from public schools had a 63% reduced chance of acquiring HPV infection when compared to the participants who came from public schools (OR: 0.3713, *p* = 0.0035), because young women who studied in public schools in previous years are low-income and suffer the impacts of socioeconomic fragility and difficulty in receiving medical care and accepting vaccinations, either because they are from peripheral populations of the capital or from remote communities [54].

The limitations of this study are the low sample size obtained mainly among students who came from cities in the interior of the state of Para. We did not investigate the circulating genotypes of both infections, which made it impossible for us to perform epidemiological analyses according to the genotypic distribution pattern to attempt any comparison with the cytological conditions. The low student participation rate is possibly due to a lack of awareness among students, fear or stigma associated with participating in an STI study, or limited access to health care due to socioeconomic barriers. The sampling bias is possible because the university students answered the questionnaire according to socially shaped measures, distorting the true condition of the socioepidemiological indicators of the infection.

## 5. Conclusions

Endocervical infection by *C. trachomatis* was significantly present in college women who had a family income below the Brazilian minimum wage and students under 25 years old. Women who did not have children were twice as likely to acquire endocervical HPV infection, and participants who did not study in a public school during basic education had a significantly reduced risk of HPV infection. Further studies will be important to understand the distribution of *C. trachomatis* and HPV genotypes and their clinical implications in Amazonian populations. STI prevention policies need to be intensified for the female population of young university students in the Amazon region to understand the epidemiological patterns of these infections and ensure future control and prevention of secondary pathologies such as PID and cervical cancer.

## Figures and Tables

**Table 1:** Comparison of the prevalence of endocervical infection by *C. trachomatis* and HPV in college women from the capital (Belem) and the interior of the state of Para, Amazon, Brazil.

Prevalence by Municipality of Origin	Total (*n* = 302)		*C. trachomatis* (+) (N = 25)		OR	CI (95%)	*p*-Value	HPV (+) (N = 87)		OR	CI (95%)	*p*-Value
	N	%	N	%	2.9259	0.8517–10.0521	0.1226	N	%	1.1457	0.6481–2.0188	0.7484
Interior	82	27.2	3	3.5				22	26.8			
Capital	220	72.8	22	10				65	29.6			

Descriptive data on the prevalence of *C. trachomatis* and HPV infection. OR: Odds ratio. CI (95%): 95% confidence interval. *C. trachomatis* (+): *C. trachomatis* positive. HPV (+): HPV-positive. n: Total number of the study population; N: Number of the specific stratified population for both *C. trachomatis* positive and HPV positive cases.

**Table 2:** Prevalence, socioepidemiological characteristics, and sexual behavior of college women in the state of Pará, Amazon, Brazil.

Variables		Total (*n* = 302)		CT (+) N = 25 (8.3%)		OR	CI (95%)	*p*-Value	*p*-Value ±	HPV (+) N = 87 (28.9%)		OR	CI (95%)	*p*-Value	*p*-Value ±
		N	%	N	%					N	%				
**Age (years)** ^a^	<25	145	48	15	10.3	1.6472	0.7155–3.7945	0.3301	0.2342	57	39.3	2.6989	1.6054–4.5371	0.0002 *	0.0049 *
	≥25	157	52	10	6.4					30	19.1				
**Marital status** ^a^	Single	274	90.7	22	8.1	0.6640	0.1847–2.3873	0.7958	0.1205	82	29.9	1.7938	0.6840–5.1063	0.3103	0.7886
	Married	28	9.3	3	10.7					5	17.8				
**Family income** †^c^	<1	54	17.9	8	14.8	0.119 †	-	-	0.0497 *	14	25.9	0.022 †	-	-	0.6598
	1–3	218	72.2	16	7.3					62	28.5				
	>3	30	9.9	1	3.3					11	36.6				
**Children** ^b^	No	242	80.1	22	9.1	1.9000	0.5493–6.5720	0.4426	0.1842	77	31.8	2.3333	0.1235–4.8461	0.0307 *	0.0482 *
	Yes	60	19.9	3	5					10	16.6				
**Alcohol** ^a^	Yes	183	60.6	9	4.9	0.8540	0.3645–2.0008	0.8808	0.6584	32	17.4	0.8560	0.5120–1.4310	0.6432	0.6826
	No	119	39.4	16	13.4					55	46.2				
**From public school** ^a^	Yes	254	84.1	19	7.5	0.5345	0.2012–1.4199	0.3254	0.1968	65	25.6	0.3713	0.1951–0.7064	0.0035 *	0.0178 *
	No	48	15.9	6	12.5					22	45.8				
**Age of first intercourse (years)**	≤15	70	23.8	5	7.1	0.8154	0.2944–2.2581	0.8840	0.4112	21	30	1.0779	0.6003–1.9356	0.9198	0.6768
	>15	232	76.2	20	8.6					66	28.4				
**Number of sexual partners in life** ^b^	1	48	15.9	2	4.2	0.6068	0.1385–2.6593	0.7234	0.6245	12	25	0.7956	0.3924–1.6129	0.6445	0.9309
	>1	254	84.1	23	9.1					75	29.5				
**Constant condom** **use** ^b^	Yes	82	27.1	5	6.1	1.4851	0.5381–4.0989	0.5953	0.4771	24	29.2	0.9245	0.5280–1.6188	0.8961	0.8909
	No	220	72.9	20	9.1					63	28.6				
**Miscarriage** ^b^	Yes	34	11.3	4	11.8	0.5323	0.1760–1.6026	0.3692	0.1148	10	29.4	1.0335	0.4720–2.2631	0.7635	0.4407
	No	268	88.7	21	7.8					77	28.7				
**Menarche** ^b^	≤15	292	96.7	24	8.2	0.7138	0.0856–5.9488	0.7635	0.8747	86	29.4	3.3237	0.4094–26.9805	0.4142	0.8620
	>15	10	3.3	1	10					1	10				

CT: *C. trachomatis*. ^a^ Chi-square test, ^b^ Fisher’s exact, ^c^ G test. † Equivalent to Brazilian minimum wage. OR: Odds Ratio. CI (95%): ± Multiple Logistic Regression. † Cramer’s V test for variables with more than two lines considering level of association as weak (≤0.29), moderate (0.3–0.49) or strong (≥0.5). Confidence interval of 95%. CT (+): *C. trachomatis* positive. HPV (+): HPV-positive. *: statistically significant *p*-value. n: Total number of the study population; N: Number of the specific stratified population for both *C. trachomatis* positive and HPV positive cases.

**Table 3:** Pap smear results of college women from the state of Para, Amazon, Brazil.

Cytological and HPV Vaccination Conditions		Total (*n* = 302)		CT (+) (N = 25)		OR	CI (95%)	*p*-Value	HPV (+) (*n* = 87)		OR	CI (95%)	*p*-Value
		N	%	N	%				N	%			
Cytological conditions	LSISL/ASCUS	19	6.3	2	10.5	-	-	0.9920 ^G^	7	36.8	-	-	0.1418 ^G^
	HSIL/ ASC H	11	3.6	1	9.1				6	54.5			
	Normal	272	90.1	22	8.1				74	27.2			
Pap smear annually	Yes	215	71.2	20	9.3	1.6821	0.6106–4.6339	0.4326	63	29.3	1.0881	0.6250–1.8940	0.8745
	No	87	28.8	5	5.7				24	27.5			
HPV Vaccination	Yes	80	26.5	-	-	-	-	-	28	35	1.4876	0.8604–2.5720	0.1997
	No	222	73.5	-	-	-	-	-	59	26.6			

CT: *C. trachomatis.* OR: Odds Ratio. ^G^: G test. CI (95%): Confidence interval of 95%. CT (+): *C. trachomatis* positive. HPV (+): HPV-positive. n: Total number of the study population; N: Number of the specific stratified population for both *C. trachomatis* positive and HPV positive cases.

## Data Availability

The original contributions presented in this study are included in the article. Further inquiries can be directed to the corresponding author.
